# Joint axes of rotation and body segment parameters of pig limbs

**DOI:** 10.1186/1751-0147-49-20

**Published:** 2007-09-06

**Authors:** Vivi M Thorup, Frede Aa Tøgersen, Bente Jørgensen, Bente R Jensen

**Affiliations:** 1 Department of Animal Health, Welfare and Nutrition, Faculty of Agricultural Sciences, University of Aarhus, Research Centre Foulum, Blichers Allé 20, PO Box 50, DK-8830 Tjele, Denmark; 2 Department of Exercise and Sport Sciences, Faculty of Science, University of Copenhagen, Panum Institute/IFI, Blegdamsvej 3, DK-2200 Copenhagen N, Denmark; 3 Department of Genetics and Biotechnology, Faculty of Agricultural Sciences, University of Aarhus, Research Centre Foulum, Blichers Allé 20, PO Box 50, DK-8830 Tjele, Denmark

## Abstract

To enable a quantification of net joint moments and joint reaction forces, indicators of joint loading, this study aimed to locate the mediolateral joint axes of rotation and establish the body segment parameters of the limbs of pigs (*Sus scrofa*). To locate the joint axes of rotation the scapulohumeral, humeroradial, carpal complex, metacarpophalangeal, coxofemoral, femorotibial, tarsal, and metatarsophalangeal joints from 12 carcasses were studied. The joints were photographed in three positions, bisecting lines drawn at fixed landmarks with their intersection marking the joint axes of rotation. The body segment parameters, i.e. the segment mass, center of mass and moment of inertia were measured on the humerus, radius/ulna, metacarpus, forepastern, foretoe, femur, tibia, metatarsus, hindpastern, and hindtoe segments from five carcasses. The segments were weighed, and their center of mass was found by balancing them. The moments of inertia of the humerus, radius/ulna, femur and tibia were found by rotating the segments. The moments of inertia of the remaining segments were calculated. Generally, the joint axes of rotation were near the attachment site of the lateral collateral ligaments. The forelimb, with segments taken as one, was significantly lighter and shorter than the hindlimb (P < 0.001). In all segments the center of mass was located 31 to 50% distal to the proximal segment end. The segment mass decreased with distance from the trunk, as did the segment moment of inertia. The results may serve as reference on the location of the joint axes of rotation and on the body segment parameters for inverse dynamic modeling of pigs.

## Findings

Net joint moments and joint reaction forces can be quantified using inverse dynamic modeling [1,2], provided that knowledge of the body segment parameters (BSPs) and the locations of the joint axes of rotation (JARs) exists. BSPs are required as input for the inverse dynamic model, and JARs define the boundaries of the model segments. To the best of our knowledge neither BSPs nor JARs have been studied in pigs, therefore this study aimed to locate the mediolateral JARs and establish the BSPs of segments from fore- and hindlimbs of healthy pigs.

**Figure 1:**
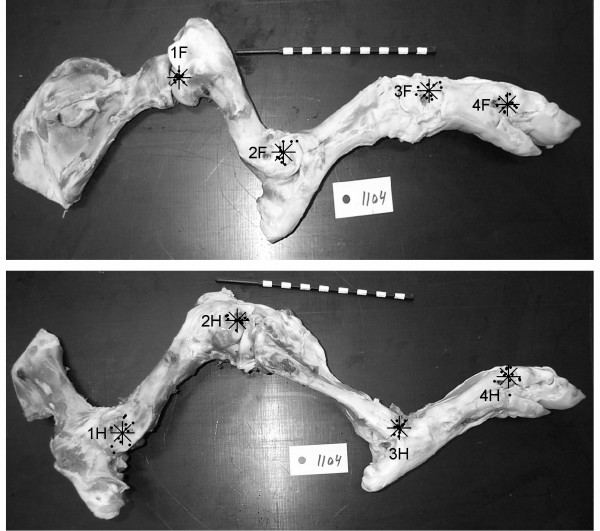
**The joint axes of rotation of the pigs' limbs**. The fore- and hindlimbs with the average (crosses) and individual JARs (dots) of 12 pigs related to one animal. Top: Forelimb with the shoulder (1F), elbow (2F), carpal (3F) and fetlock (4F) JARs. Bottom: Hindlimb with the hip (1H), stifle (2H), hock (3H) and fetlock (4H) JARs. The lateral side of the bones is up. For scaling purposes a measuring stick with black and white fields of 1 cm was placed next to the bones.

To locate the JARs 12 Duroc-Yorkshire-Landrace crossbred (D(YL)) pigs were studied: six castrates and six gilts without clinical limb abnormalities. Their body weight (BW) at slaughter was 77 ± 7 kg. Right fore- and hindlimbs were removed without disarticulating the joints. The eight joints examined were the: scapulohumeral (shoulder, 1F); humeroradial (elbow, 2F); carpal complex (carpal, 3F); metacarpophalangeal (forefetlock, 4F); coxofemoral (hip, 1H); femorotibial (stifle, 2H); tarsal (hock, 3H) and metatarsophalangeal joint (hindfetlock, 4H) (Fig. 1). With the bones lying on the medial side digital photos were taken of each joint in extended, neutral and flexed position around the mediolateral axis. JARs were calculated according to the Realeaux-technique previously applied to the equine limbs [3]. The photos were aligned by two distinct landmarks on one bone of the joint. On the other bone the JAR was located as the intersection of the mid-perpendicular lines of the displacement vectors of two distinct landmarks at consecutive joint positions. Usually, three points of intersection were generated therefore an arithmetic average of the points was calculated. Results were described qualitatively in relation to bony landmarks palpable on the skin surface. Measured on a test object (five measurements of three JAR positions repeated on two days) the JAR technique absolute error was 0.31 ± 0.09 cm, calculated as the mean distance of the estimated JARs from the known JARs. The variable error was 0.05 ± 0.03 cm, calculated as the mean distance between pairs of the estimated JARs. An ANOVA revealed no significant differences between days, neither in absolute error (F = 2.63; P = 0.14) nor in variable error (F = 1.60; P = 0.24).

To establish the BSPs five D(YL) crossbred pigs were used: one castrate and four gilts without clinical limb abnormalities. Their live BW was 69 ± 5 kg. After exsanguination the right fore- and hindlimbs were separated from the trunk and cooled lying horizontally. The day after slaughter the carcasses including limbs were weighed. Blood and water loss summed to 5.2 ± 0.2% BW. The chilled limbs were dissected into segments along cranio-caudal lines running through the JARs identified above. The ten segments investigated were the: humerus; radius/ulna; metacarpus; forepastern (proximal and middle phalanges); foretoe (distal phalanges); femur; tibia; metatarsus; hindpastern; and hindtoe. The segments were frozen lying horizontally. Mass, length, distance (d_prox_) from center of mass (COM) to proximal segment end, and moment of inertia (hereafter referred to as inertia) were measured on the frozen segments. Sagittal plane COM was located by balancing the segments transversely and longitudinally on a sharp edge. A line of balance was drawn in each direction, the intersection thus marking the COM. The relative position of the COM (COM_rel_) was calculated as the d_prox _in percent of segment length. The inertia was measured by strapping the segments onto a custom made low-friction horizontal turntable; an external load connected to the turntable was dropped and turned the turntable. The external load passed between two photocells. Photocell data were converted (Data Translation 9800 A/D converter) and sampled at 1 kHz, thus measuring drop time. The inertia was calculated from load drop time (t_l_) according to formula 1:

inertia = (m_l_·g·r_t_^2^·t_l_^2^)/2s_p_

in which external load mass (m_l_) was 0.203 kg, gravitational acceleration (g) was 9.82 m/s^2^, turntable radius (r_t_) was 0.15 m, and distance between photocells (s_p_) was 1.317 m. Segment inertia was calculated by subtracting the inertia of the unloaded turntable from the inertia of the loaded turntable. The humerus, radius/ulna, and tibia were placed with the proximal segment end aligned with the turntable center, so these inertias around the proximal segment end (I_prox_) were converted to inertias around the segment COM (I_COM_) using the parallel-axes theorem in formula 2:

I_COM _= I_prox _- m_s_·d_prox_^2^

where m_s _was the segment mass. The femur was placed with the COM at the turntable center and no conversion was necessary. The metacarpus, forepastern, metatarsus, and hindpastern were too light (< 0.3 kg) to have their inertia measured, thus their I_COM _was estimated from circumference and length [4] according to formula 3:

I_COM _= m_s_/12·(length^2 ^+ 0.076·circumference^2^)

assuming cylindrical segments. Mass, length and d_prox _were measured once on five animals, whereas circumference was measured once on three animals. Load drop time for the unloaded turntable and for each segment was measured six times from which individual means were calculated. Results were reported as group average with standard deviations. Paired t-tests were performed to compare differences in segment mass, length and inertia for the fore- and hindlimbs. Level of significance was 5%.

The shoulder JAR was on the humerus' head, near the posterior part of the greater tubercle. The elbow JAR was mainly located on or around the lateral condyle of the humerus, where the lateral collateral ligament is attached. The rotation axis of the carpal joint complex was mostly on and around the fourth carpal bone, on which the accessorioquartale ligament is attached. The forefetlock JAR was located around the most distal part of the fourth metacarpal bone, slightly distal and posterior to the attachment site of the lateral collateral ligament. The hip JAR was located posteriorly on the greater trochanter. The stifle JAR was just distal and anterior to the femur's lateral condyle, the attachment site of the lateral collateral ligament. The hock JAR was located around the attachment site of the lateral collateral ligament on the fibula's lateral malleolus. The hindfetlock JAR was distal to the lateral condyle on the fourth metatarsal bone. The 12 individual JARs and their averages scaled to the fore- and hindlimb of one randomly chosen pig are shown in Fig. 1.

For palpation purposes the JARs were mainly at or near the attachment site of the lateral collateral joint ligaments, thus allowing movements without excessive ligament strain. The JAR locating method assumed that all joints were revolute, however the spread locations of JARs suggested that, for instance in the hip and stifle joints, slight cranio-caudal translation may also have occurred. Besides, the removal of muscle and skin to expose bony landmarks and to avoid skin movement errors may have allowed the joints to deviate slightly from their anatomical sagittal plane. Nevertheless, large joint rotations were performed between consecutive positions to minimize JAR estimation errors [5,6].

Adding all limb segments the forelimb and hindlimb weighed 3.3 ± 0.2% BW and 8.6 ± 0.2% BW, respectively; the forelimb length was 40.6 ± 1.5 cm and the hindlimb measured 52.9 ± 1.6 cm, thus the forelimb was significantly lighter and shorter than the hindlimb (P < 0.001). These differences were mainly caused by the relatively heavy and long femur, tibia and metatarsus (Table 1). The COM_rel _was in the proximal part of all segments. Segment mass and inertia decreased with increasing distance from the trunk, thus proximal segments were the heaviest and had the largest inertias.

**Table 1:** The body segment parameters of the right limbs of five pigs. The segment mass, kg and % BW; segment length, cm; segment COM_rel_, the distance from the proximal segment end to the COM in % of segment length; and segment I_COM_, kg·m^2^·10^-3^, are presented as average ± s.d.

	**Mass**		**Length**	**COM_rel_**	**I_COM_**
	
	kg	% BW	cm	%	kg·m^2^·10^-3^
**Forelimb**					
Humerus	1.333 ± 0.126	1.94 ± 0.12	12.7 ± 0.2	46.1 ± 1.9	4.42 ± 1.07
Radius/ulna	0.726 ± 0.073	1.05 ± 0.04	14.5 ± 1.2	31.5 ± 3.0	2.32 ± 0.70
Metacarpus	0.125 ± 0.021	0.18 ± 0.03	6.4 ± 0.8	49.3 ± 2.1	0.06 ± 0.03^b^
Forepastern	0.100 ± 0.008	0.15 ± 0.01	4.9 ± 0.1	44.5 ± 2.1	0.04 ± 0.00^b^
Foretoe	0.030 ± 0.003	0.04 ± 0.00	2.2 ± 0.1	50^a^	0.0002^a^

**Hindlimb**					
Femur	4.466 ± 0.207	6.50 ± 0.22	18.3 ± 1.0	50.3 ± 5.1	31.50 ± 2.37
Tibia	0.991 ± 0.056	1.44 ± 0.07	16.0 ± 0.9	40.4 ± 3.6	2.52 ± 1.00
Metatarsus	0.291 ± 0.035	0.42 ± 0.03	10.4 ± 0.8	32.3 ± 5.6	0.34 ± 0.07^b^
Hindpastern	0.111 ± 0.010	0.16 ± 0.01	5.9 ± 0.6	40.0 ± 5.5	0.06 ± 0.01^b^
Hindtoe	0.029 ± 0.003	0.04 ± 0.01	2.3 ± 0.1	50^a^	0.0002^a^

^a^approximated; ^b^calculated.

The BW of the pigs in the BSP study varied 7% between individuals, whereas the BSPs varied more, e.g. the inter-individual coefficient of variations of the measured inertia were: humerus 14%; radius/ulna 31%; femur 7% and tibia: 28%. These variations were in line with those reported for horses [7,8] and dogs [2]. Although the dissection procedure was performed by the same experienced technician this may have contributed to the variation. Furthermore the variation between body segments from pigs of similar BW may be explained by conformation differences, e.g. the large variation of the metacarpus was mainly caused by a very short (5.0 cm) and light (0.091 kg) segment in one pig.

The COM and inertia of the toe segments were approximated, as these segments could not be balanced and were too light to have their inertia measured. However considering their small masses, their inertia will be negligible therefore it was approximated as the lowest reasonable input value for the inverse dynamics model, based on resolution limits. In inverse dynamics the inertias are used for calculating net joint moments only, and during the stance phase contributions from inertial parameters to net joint moments are very small because the angular accelerations of the limb segments are low [4]. Furthermore measuring the BSPs on exsanguinated and frozen segments resulted in lower masses due to the 5.2% BW blood loss and water evaporation. However the distribution of blood and water cannot be assumed to be uniform across segments, because distal segments have a higher bone to muscle ratio and thus less blood than proximal segments, which should be accounted for in inverse dynamic modeling.

This investigation offers the first experimental data on the JARs and BSPs of pigs' limbs, thus enabling a quantification of net joint forces and moments.

## Competing interests

The author(s) declare that they have no competing interests.

## Authors' contributions

VMT participated in the study design, carried out the experiments and drafted the manuscript. FAT calculated the JAR locations. BJ and BRJ designed the experiments and helped drafting the manuscript. All authors read and approved the final manuscript.
